# Transcriptomic Profile of Isocitrate Dehydrogenase Mutant Type of Lower-Grade Glioma Reveals Molecular Changes for Prognosis

**DOI:** 10.3390/biomedicines13092263

**Published:** 2025-09-14

**Authors:** Seong Beom Cho

**Affiliations:** Department of Biomedical Informatics, College of Medicine, Gachon University, Incheon 21565, Republic of Korea; sbcho1749@gachon.ac.kr

**Keywords:** lower-grade glioma, transcriptomics data, Isocitrate dehydrogenase, molecular mechanism, drug repurposing

## Abstract

**Background/Objectives:** Lower-grade glioma (LGG) is a type of brain tumor with a relatively better prognosis than glioblastoma. However, identifying therapeutic targets for LGGs remains elusive. To uncover the molecular features of LGGs, functional genomics data have been investigated. **Methods**: Using public transcriptomics data of LGGs (The Cancer Genome Atlas and GSE107850), differentially expressed genes (DEGs) and differentially co-expressed (DCE) gene pairs between IDH mutation statuses were determined. Gene set enrichment analysis identified the molecular mechanisms of isocitrate dehydrogenase (IDH) mutation in LGGs. Furthermore, the identified DEGs and DCE gene pairs were used for drug repurposing analysis. **Results**: Two public datasets revealed an overlap of 1527 DEGs. Whereas only seven gene pairs showed significant differential co-expression in both datasets, 1016 genes were simultaneously involved in differential co-expression. Gene set enrichment revealed that biological processes related to neuronal tissue formation were significantly associated with the DEGs. Using drug repurposing analysis, it was found that NVP-TAE684 and bisindolylmaleimide were possible chemical compounds for the LGG treatment. **Conclusions:** Using transcriptomics data, molecular mechanisms associated with LGG prognosis were identified. This work provides clues for future research on LGG treatment.

## 1. Introduction

Lower-grade gliomas (LGGs) are a subtype of brain tumors originating from glial cells, which normally have supportive roles for neurons [1]. These tumors show slower growth and lower malignancy potential than high-grade gliomas. Even though they grow slowly, by compressing the neighboring brain regions, LGGs can produce significant symptoms, including headache, vision disturbances, and seizures [2,3].

Isocitrate dehydrogenase (IDH) mutations are a hallmark of LGGs, and they are less frequent in glioblastoma, a more aggressive form of brain tumor [4,5]. Mutations occur more frequently in the *IDH1* gene than in the *IDH2* gene, producing 2-hydroxyglutarate (2-HG), an oncometabolite that alters cellular metabolism [6]. The IDH mutation subgroup of patients shows better prognosis and treatment response than patients with the wild-type IDH [7]. This indicates that molecular changes induced by IDH mutations are critical to the survival of glioma patients. Notably, the IDH mutations inhibit enzymes involved in DNA and histone demethylation, resulting in widespread epigenetic programming and gene expression changes [8,9]. Genes involved in differentiation and immune response were found to be downregulated in IDH-mutant gliomas [10], while OLIG2, which is expressed in the normal adult brain, was overexpressed in gliomas [11]. Moreover, IDH mutation is one of the key factors considered in the grading of diffuse astrocytic gliomas [12]. Considering this, information about such changes can provide clues for the management of LGGs.

Previous studies revealed altered gene expression profiles between wild-type and mutant IDH groups. Wu et al. identified metabolic genes that were differentially expressed between the IDH status groups of LGG [13]. They showed that *ACAA2*, which is involved in fatty-acid beta-oxidation, was differentially expressed between the subgroups and associated with prognosis. Using transcriptome data of 24 patients, Li et al. revealed that phospholipase Cγ1 (*PLCG1*) expressions were associated with clinical outcomes of LGGs [14]. siRNA for *PLCG1* substantially impacted the pathologic behavior of IDH wild-type LGG cell lines. Immunogenic cell death-related genes had predictive potential for prognosis and sensitivity to immune checkpoint blockade therapy [15]. These results indicate that transcriptional changes between IDH status groups provide invaluable information for estimating molecular mechanisms related to prognosis or therapeutic targets.

Given these observations, the transcriptome data of the Cancer Genome Atlas (TCGA) lower-grade glioma (LGG) [16] and GSE107850 [17] were analyzed based on the IDH status. While previous research using the same datasets focused on identifying novel clusters with different prognoses or classifications, or on finding biomarkers for LGG prognosis, this study investigated molecular mechanisms related to IDH mutations and their potential applications in the treatment of LGGs. Using the differentially expressed genes (DEGs) and differentially co-expressed (DCE) gene pairs, molecular mechanisms of prognostic changes between IDH status were identified. Drug repurposing analysis was also performed with the genes and gene pairs. The overall analysis flow is presented in Figure S5.

## 2. Materials and Methods

### 2.1. Differentially Expressed Gene Analysis

Differentially expressed gene (DEG) analysis involves the determination of genes that show significant differences in their expression between different conditions. The Wilcoxon rank-sum test was applied to identify DEGs between the IDH mutation and wild-type groups. Although there are many methods for DEG analysis, the rank-based method was applied to determine more robust results, even at the sacrifice of sensitivity. After determining *p*-values, multiple testing corrections, including Bonferroni and Benjamini–Hochberg (BH) methods, were considered according to the number of significant results.

### 2.2. Differential Coexpression of Gene Pairs Between IDH Status

Differential co-expression (DCE) analysis identifies significant changes in co-expression patterns between conditions. DCE analysis was applied to reveal changes in gene expression regulation. The DCE analysis tests the significant changes in co-expression between different conditions. For this purpose, co-expressions between genes are measured, and Fisher transformation (Equation (1)) is applied to each condition. The co-expressions are determined by the Pearson correlation coefficient (PCC) between the expression vectors of the gene pair.

(1) $$z=\mathrm{In}\frac{\left(1+PCC\right)}{\left(1-PCC\right)}$$

The co-expression difference was tested on the standard normal distribution using the normalized difference between the PCCs (Equation (2)). In Equation (2), *N*_1_ and *N*_2_ indicate the number of samples in different groups.

(2) $$Z=\frac{\left(Z_{1}-Z_{2}\right)}{\sqrt{\left(\frac{1}{N_{1}-3})+(\frac{1}{N_{2}-3}\right)}}$$

### 2.3. Gene Set Enrichment Analysis Using Gprofiler2

After DEG and DCE analysis, gene set enrichment analysis was applied to reveal possible molecular mechanisms. The gprofiler2 R package (version 0.2.3) was used for the enrichment analysis [18]. The package contains various annotation databases, including Gene Ontology (GO) [19], Kyoto Encyclopedia of Genes and Genomes (KEGG) [20], Reactome [21], WikiPathways [22], and Human Phenotype Ontology [23]. These databases provide gene sets with a wide range of biological implications. Multiple testing corrections for false discovery rates (FDRs) are implemented in the package, which investigates significant genes from DEG and DCE to determine if there are any biological implications based on previous knowledge provided by the databases. After the determination of significant results, a graphical representation is generated.

### 2.4. Network-Based Clustering of Genes with Protein-Protein Interaction Network

The DEG analysis is useful in identifying underlying biological mechanisms in the phenotype. However, the analysis gives no direct information about regulatory relationships between genes. On the other hand, the DCE analysis does not provide information about differential gene expression. Therefore, overlapping genes in the DEG and DCE analysis results may reveal core biological processes related to the pathophysiology of IDH mutations. Moreover, closely connected genes of the overlapping genes might be a representative core process. To identify such clusters of genes, overlapping genes from the results of DEG and DCE analysis were applied to Markov clustering (MCL). Markov clustering (MCL) is a graph-based clustering algorithm that identifies strongly connected nodes and partitions them into clusters. The genes were applied to the STRING web tool [24], where the MCL was implemented for the analysis. The MCL was performed with default parameters.

### 2.5. Drug Repurposing Analysis with L1000 Data and CLUE Platform

For the discovery of therapeutic chemicals or drugs that had already been used for the treatment of other diseases or investigated in preclinical studies, a connectivity map (CMap) method and CLUE platform were applied. The CMap method uses the rank of DEGs and is organized to find drugs or chemicals that show the reverse rank of the DEGs compared with that of diseases [25]. The method was implemented in the CLUE platform (https://clue.io, accessed on 1 May 2025), a cloud-based software system for CMap analysis. The DEGs of diseases are determined by comparing gene expressions between normal and disease groups. Transcriptomics data from pre- and post-treatment drugs or chemicals are used to determine DEGs induced by the compounds. This method assumes that if the rank of DEGs associated with the administration of a compound is reversed compared to the rank of DEGs from the comparison between normal and disease samples, the compound would have therapeutic effects. The CLUE platform and the L1000 dataset were used to identify therapeutic compounds with DEGs from this analysis [26].

## 3. Results

### 3.1. Lower-Grade Glioma Data from Public Databases

For this research, two LGG datasets were used. The first was retrieved from CBioportal, which provides multi-omics datasets of various types of cancers [27]. From the website, LGG multi-omics datasets that were produced by TCGA project were used in the analysis [28]. The dataset comprises clinical data, including age, sex, survival length information, and molecular markers, including IDH mutation status. Of the patients, 415 had mutations in IDH. The transcriptomics data with 508 samples were also produced using the Illumina sequencing platform, and gene expression was estimated using the RSEM method [29]. The 20,511 genes were included in the TCGA LGG data. The clinical information of the two datasets was summarized in Supplementary Results (Table S1).

Another LGG dataset was retrieved from the GEO database. Although LGG transcriptomics data were available, GSE107850 data were used because IDH mutation information was provided. The data were generated with the Illumina microarray platform, which has 29,377 probes for transcripts. The IDH mutation information and data matrix were extracted from the series matrix file of GSE107850. The mapping of probes to gene symbols was performed using the Illumina R package [30]. The data contained 180 samples, and the number of IDH mutation cases was 166.

In the preparation of RNA samples from the TCGA dataset, an AllPrep kit (Qiagen, Venlo, The Netherlands) was used for tissue samples [31]. IDH mutation status was defined based on the presence of mutations in either *IDH1* or *IDH2*. For the GSE107850 dataset, RNA was isolated from formalin-fixed, paraffin-embedded tissue blocks or snap-frozen tissue samples. Purified RNA (250 ng) was applied to DASL beadchips (Illumina, San Diego, CA, USA). Quantile normalization and batch correction were performed using the preprocessCore (Bioconductor) and ber (R) packages, respectively [17].

### 3.2. Differential Expression Between IDH Status Groups

When the Wilcoxon rank-sum test was applied to the TCGA LGG data, 7970 genes showed significant differential expression between IDH status groups (Bonferroni-adjusted *p*-value = 2.44 × 10^−6^). Of these genes, 3408 were upregulated, and 4562 were downregulated in the IDH mutant group (Table S2). In the GSE107850 data, 22 genes had significant expression changes between IDH status groups (Bonferroni-adjusted *p*-value = 1.70 × 10^−6^, Table S3). Two of these genes (*RPLP1* and *CALCRL*) were upregulated, and the remaining 20 were downregulated in the IDH mutant group.

**Figure 1:**
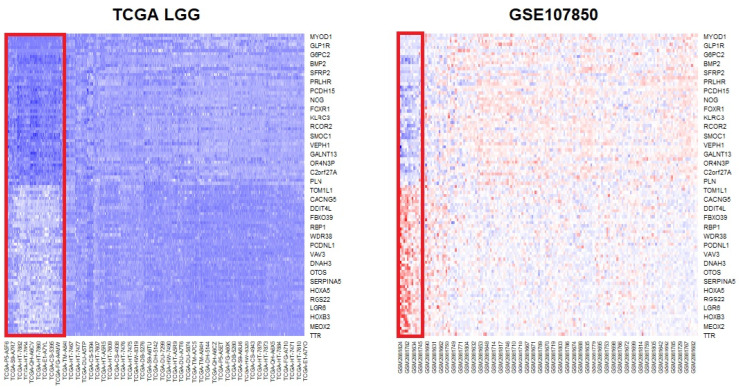
Heatmaps of the top 50 up- and downregulated genes from the differential expression analysis (DEG) of IDH status groups. The gene rank was determined based on the TCGA data. The upper part of the heatmaps shows upregulated genes, while the lower part shows the expression of downregulated genes in the IDH mutation status group. The red rectangle indicates the wild-type IDH group. On the TCGA heatmap, dark blue indicates downregulation, whereas on the GSE107850 heatmap, bright blue and red indicate downregulation and upregulation, respectively. DEG: differentially expressed genes; IDH: isocitrate dehydrogenase.

To robustly identify DEGs, common genes that appeared in the results of the two datasets and that had the same direction of mean expression changes (up- and downregulation) were selected. Since the number of DEGs was relatively small because of the GSE107850 data, BH multiple testing correction was applied to relax the *p*-value threshold and determine more DEGs. The p.adjust R function was applied for BH correction, and 2845 genes were found to be significant (adjusted *p*-value < 0.05). In total, 1472 genes were upregulated, and 1373 were downregulated. The common DEGs were determined from the two significant DEG lists, and 1526 genes overlapped (Table S4). Of these, 697 genes were upregulated and 829 genes were downregulated. The most significantly upregulated gene was *MYOD1*, and *ADAMTS20*, *LHX5*, *GLP1R*, and *NDST4* were top-ranked genes. *TTR* was the most significantly downregulated gene, and *STAC*, *APCDD1L*, *MEOX2*, *SPAG17*, and *C6orf15* were also highly downregulated in the TCGA LGG data (Figure 1 and Table S4).

**Figure 2:**
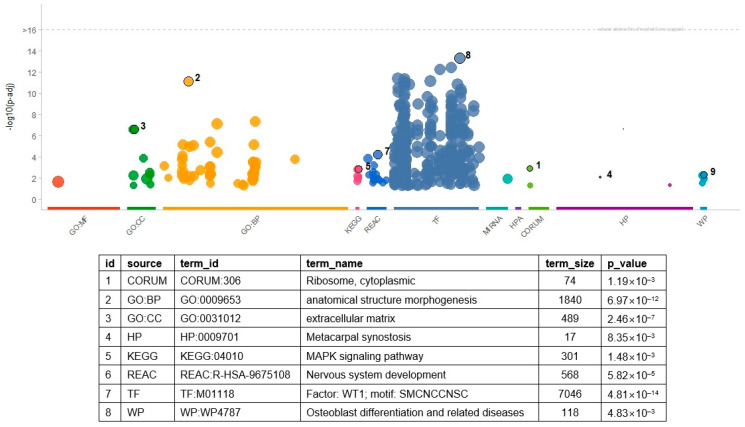
Result of gene set enrichment analysis with DEGs. The numbers indicate the most significant result in a category of gene set. In the table, source is the category of the gene sets, and term_id and term_name indicate the identifier of a gene set and its name. The *p*-values of the table are determined in terms of the false discovery rate. The numbers in the table indicate the most significant terms in each category. Table S5 includes the full list of the significant GO terms and related information. GO: gene ontology, BP: biological process, CC: cellular compartment, HP: human phenotype ontology, KEGG: Kyoto Encyclopedia of Genes and Genomes, REAC: Reactome, TF: transcription factor, WP: wiki pathway.

Enrichment analysis revealed many molecular processes and pathways (Figure 2 and Table S5). In total, 377 gene sets were significant (with an FDR threshold < 0.05), including 293 transcription factor (TF) gene sets. The gene set of *WT1* showed the most significant result of the TF gene sets (*p* = 4.81 × 10^−14^). In the *WT1* gene set, 670 genes—including *ABCC8*, *CACNA1A*, and *SMAD9*—overlapped with the input genes. Thirty-nine GO BP terms were significant, and the hierarchically top-ranked terms in the GO network showed highly significant results. For example, ‘anatomical structure morphogenesis’ (*p* = 6.97 × 10^−12^) and ‘anatomical structure development’ (*p* = 4.50 × 10^−8^) showed the first and second most significant results. In addition, more specialized processes such as ‘cell adhesion’ (*p* = 6.84 × 10^−6^), ‘cell mobility’ (*p* = 8.20 × 10^−4^), ‘neuron differentiation’ (*p* = 7.71 × 10^−4^), and ‘cell migration’ (*p* = 1.71 × 10^−3^) were also significant. The functionality of several significant pathways seemed consistent with the significant GO terms. For example, the KEGG ‘PI3K-Akt signaling pathway’ and WP ‘focal adhesion PI3K Akt mTOR signaling’ related to the cell adhesion process were significant. The significance of KEGG’s signaling pathways in regulating the pluripotency of stem cells is consistent with the GO terms related to the developmental process.

### 3.3. Significant Change of Co-Expressed Gene Pairs Between IDH Status

Gene selection with a variance filter was applied to determine differential co-expression analysis. After determining the gene expression variance, genes were sorted according to the variance, and the top 25% of genes were selected for differential co-expression analysis. In the TCGA LGG data, 5128 genes were selected, and differences in z-transformed correlation coefficients were computed. With a Bonferroni-adjusted *p*-value (*p* = 3.80 × 10^−9^), 32,152 gene pairs showed significant differential co-expression.

When the variance filter was applied to GSE107850 data, 6988 genes were selected. Differential co-expression identified 41 gene pairs with a Bonferroni-adjusted *p*-value (*p* = 2.05 × 10^−9^). Because no overlapping gene pairs were used in the TCGA LGG data, BH multiple testing correction was applied to relax the *p*-value threshold and increase the possibility of finding overlapping gene pairs (*p* = 1.71 × 10^−5^). When the BH method was applied, 8360 gene pairs showed significant co-expression changes between IDH status groups. With the BH-corrected *p*-value, only seven gene pairs overlapped between the two results (Table 1).

**Figure 3:**
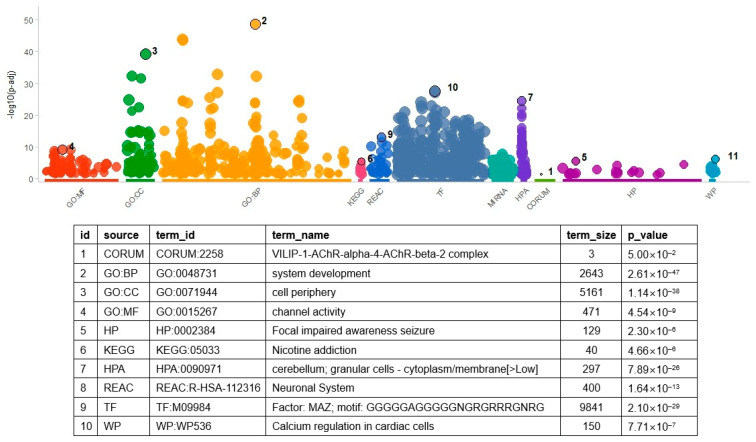
Result of gene set enrichment analysis with result genes from DCE analysis. As shown in the figure, the number of gene set categories increased. Variable names in the above table were the same as those in Figure 2. For the identification of related genes and GO terms, refer to Table S6. Acronyms in the table are the same as those in Figure 2.

In addition to determining overlapped gene pairs, common genes in the differential co-expression analysis results of the two datasets were determined to find molecular themes. Considering all analyzed genes, 1016 overlapped. These genes were used as input to the gene set enrichment analysis. As in the enrichment analysis of DEGs, many terms (*n* = 1035) were found to be significant (Figure 3 and Table S6). The TF gene sets had the largest number of significant results (*n* = 432). MAZ TF showed the most significant result (*p* = 2.10 × 10^−29^), which indicated that genes controlled by MAZ were enriched in the DCE genes. The *MAZ* gene set had 675 genes overlapping with the input genes, including *HLA-DRA*, *NOTCH2*, and *GFAP*. The GO BP gene set had the second largest number of significant results (*n* = 274, Table S6), of which ‘system development’ was the most significant (*p* = 2.61 × 10^−47^).

Finally, to identify transcription factors (TFs) associated with changes in gene expression regulation between IDH status, TFs that were commonly significant in the enrichment analyses of DCE results from both datasets were examined. First, genes from the significant DCE results were identified and then used for enrichment analysis. After the analysis, TF terms showing significant enrichment were extracted. In the TCGA dataset, 455 TFs were found to be significant. In the GSE107850 dataset, 171 TF terms were significant. Among these, 166 TF terms were significant in both datasets (Table S7).

### 3.4. Clustering of Differentially Expressed and Co-Expressed Genes with Protein-Protein Interaction (PPI) Network

The DEG and DCE analyses identified many significant genes and gene pairs. The overlapped genes of the two analyses were selected to reveal underlying biological mechanisms involved in regulating the genes. The genes were used as an input to the STRING database. In total, 232 genes were selected from the DEG and DCE analysis results. In the STRING database, 231 genes were mapped, and more PPIs were enriched between the genes than predicted by random chance (*p* = 1.78 × 10^−15^). The enrichment analysis of the genes had 124 significant results (Table S8). General biological processes containing many genes showed highly significant results. For example, the system development GO BP term showed the most significant result (*p* = 9.18 × 10^−11^). Multicellular organism development (*p* = 2.42 × 10^−10^), anatomical structure development (*p* = 5.77 × 10^−10^), and multicellular organism process (*p* = 3.71 × 10^−7^) also showed top-ranked general processes. Besides these processes, cell adhesion (*p* = 4.37 × 10^−9^) showed highly ranked significance.

**Figure 4:**
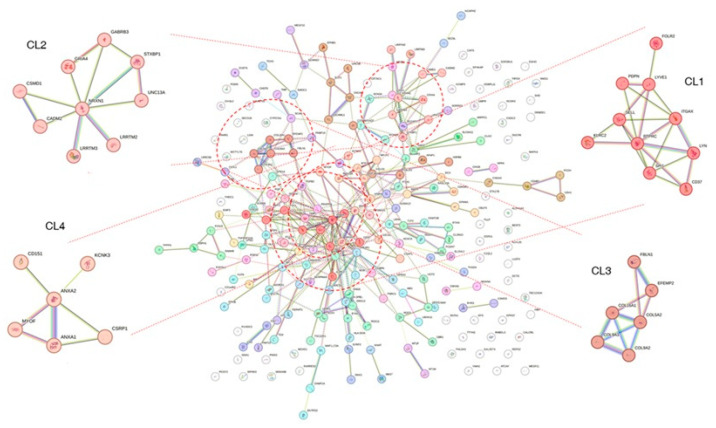
Result of Markov clustering with protein-protein interaction network. The clustering analysis was performed on the overlapping genes between the DEG and DCE analysis results. As shown in the figure, four clusters are highlighted. Note that many genes are not assigned to specific clusters and do not show links to other proteins. DEG: differentially expressed genes; DCE: differential co-expression.

The overlapping genes were also analyzed using the MCL method provided by the STRING database. Because the clustering was performed with the PPI network, the cluster determination depends on the closeness of the network structure. The MCL determined 59 clusters (Figure 4 and Table S9). Clusters with higher numbers of nodes and edges were assigned to the top clusters, and these clusters tended to show distinct biological characteristics in the enrichment test performed using the SRING database. For example, cluster1 (CL1) showed many significant GO BP terms in the enrichment analysis (Table S10). The terms were related to immune reaction or cytotoxic processes such as ‘positive regulation of leukocyte-mediated cytotoxicity’ (*p* = 0.0077), ‘positive regulation of immune system process’ (*p* = 2.60 × 10^−2^), and ‘defense response’ (*p* = 1.72 × 10^−2^). CL2 showed significant terms related to the neural synapse (Table S11), including ‘synapse organization’ (*p* = 1.60 × 10^−3^) and ‘positive regulation of synapse assembly’ (*p* = 1.45 × 10^−2^). CL3 was enriched with genes that belong to the extracellular matrix modification (Table 2 and Table S12), such as ‘extracellular matrix organization (*p* = 1.33 × 10^−7^), ‘positive regulation of cell-substrate adhesion’ (*p* = 2.22 × 10^−2^), and ‘collagen-containing extracellular matrix’ (*p* = 1.67 × 10^−7^).

### 3.5. Prediction of IDH Status Using Transcriptomics Data

Using the DEGs of the transcriptomics data, prediction analysis was performed to reveal whether the DEGs had diagnostic potential. Since the platforms of the two datasets that were used in this analysis were different (RNA sequencing versus microarray), the validation of the prediction model was performed as follows. First, using the LASSO regression model, predictor genes were selected in one dataset [32]. And, the logistic regression model with the selected genes was tested in the other dataset.

**Figure 5:**
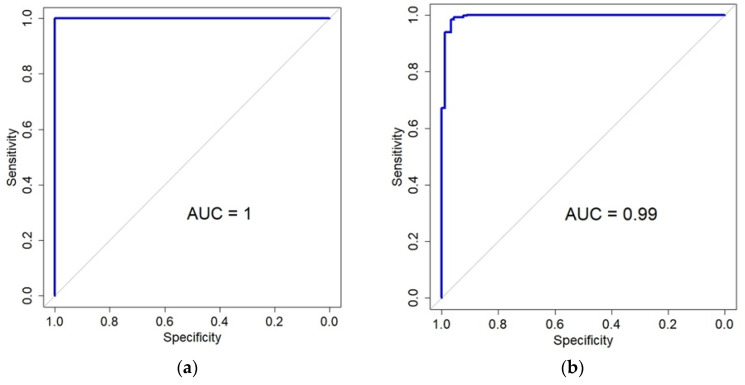
Classification performance for the prediction of IDH status using genes selected by the LASSO model. (**a**) The area under the curve (AUC) of a logistic regression model applied to the GSE107850 dataset. The 42 genes (60 probes) for this model were originally selected from TCGA data. (**b**) The AUC for a logistic regression model is 0.99 when applied to the TCGA dataset, using the eight genes that were chosen from the GSE107850 data.

In the TCGA LGG dataset, 7970 genes that were significant in the DEG analysis were used as an input to the LASSO regression model. Using the cross-validation and area under the curve (AUC), classification performance was determined. The cv.glmenet function of the glmnet R library was used with the default parameters. As a result, almost perfect performance was obtained with a minimal lambda value (mean AUC = 0.998, Figure S1). Forty-two genes were selected as predictor genes (Table S13). The genes were mapped to 60 probes of the GSE107850 dataset. When the logistic regression model was built with the genes, the AUC was also perfect (AUC = 1, Figure 5). When the LASSO model was built with the significant DEGs of the GSE107850 dataset, the mean AUC was determined to be 0.95 with the cross-validation (Figure S2). From the result, 11 genes were selected, and 8 genes were mapped to the TCGA LGG dataset. (Table S14). The logistic regression model with the 8 genes of the TCGS LGG dataset showed almost perfect performance (AUC = 0.99, Figure 5). Besides the logistic regression model, LASSO regression with cross-validation was also applied to estimate the prediction performance of the genes. When the TCGA data with 10 genes selected from GSE108750 data was applied, the mean AUC was 0.99 (Figure S3), which was equivalent to the result from the logistic regression model. However, the 60 probes selected by the TCGA data showed a mean AUC of 0.86 (Figure S4). The difference in AUC between the logistic regression and LASSO with cross-validation results seemed to stem from the unbalanced class labels of GSE108750.

### 3.6. Drug Repurposing Analysis Using CLUE Platform

To identify possible drugs that can be applied to the management of LGG, DEGs were entered into the CLUE website, and two different sets of DEGs were used for the analysis. First, the overlapped DEGs from the datasets were used for the drug repurposing analysis. Second, the genes that overlapped significantly with the results of DEG and DCE analyses were used for the repurposing analysis. Because the CLUE website allows for 150 input genes, the 150 top-ranked up- and downregulated genes of the DEGs were used for the analysis. All 232 common genes from the DEG and DCE analyses were used to input the CLUE platform.

In the repurposing analysis, drugs with high positive tau scores were selected because the IDH mutant groups show better prognosis. The up- and downregulation of the DEGs was defined according to the IDH mutation status, and a positive tau score indicated that the molecular signature of input DEGs was more similar to that of the perturbation agent (perturbagen). When the first DEG list was used, there were no drugs that showed the desired similarity (>90). However, with the second DEG list, the CLUE platform identified NVP-TAE684 (tau = 94.97) and bisindolylmaleimide (tau = 90.05).

## 4. Discussion

In this research, DEGs and DCE genes were identified in the datasets, and their possible molecular mechanisms were revealed using an enrichment analysis. While previous studies on the IDH mutation focused on detecting individual genes with significant expression changes between different IDH statuses, the current research systematically revealed a list of DEGs and DCE genes and their molecular implications. It was also revealed that the transcriptomic changes had excellent predictive power for diagnosing IDH status, which indicates that clear molecular patterns exist in the IDH mutation. Moreover, DEGs and DCE genes associated with the IDH mutation were applied to a drug repurposing analysis. Although the IDH mutation is typically used as a molecular marker in most studies, the genes of the IDH mutation were applied to drug repurposing because patients with the IDH mutation showed better prognoses.

The primary goal of this research was to identify molecular processes underlying the IDH mutation of LGGs. Because the IDH mutation is a reliable marker for the prognosis of LGGs, the molecular changes would provide information about therapeutic targets for LGGs. Based on this assumption, DEGs or DCE gene pairs were determined, and their functional implications were identified. The two datasets used in this analysis have already been utilized in other studies. However, this research focused on identifying molecular mechanisms associated with IDH status, rather than finding biomarkers or subclusters related to different prognoses. Since IDH status is closely linked to the prognosis of LGGs, understanding these mechanisms could be valuable for future research.

In this study, simple methods—Wilcoxon rank-sum test, Pearson’s correlation coefficient, and Fisher’s z-transformation—were used to identify DEGs and DCE gene pairs. Although other methods might have greater power and functionality, providing more detailed information, the chosen methods were used to obtain more robust results that are less dependent on specific platforms or data characteristics, even at the cost of reduced statistical power. The overlapping genes identified in the two datasets seemed to support this assumption. Specifically, the DEG analysis using the Wilcoxon rank-sum test showed better results in the analysis of functional genomics data compared with other sophisticated methods such as DESeq2, edgeR, limma-voom, and NOISeq [33]. This result stems from the fact that RNA sequencing data have skewed distributions, and the Wilcoxon rank-sum test is known to be well-suited for such distributions. Moreover, WGCNA, a well-known co-expression analysis method, is primarily designed to detect co-expression modules, not for the identification of gene pairs showing differential co-expression between conditions. The method is not relevant for detecting co-expression changes between different conditions.

While multiple testing correction methods were used, the results appeared to have a low probability of false positives. This is because only the genes that overlapped with the significant results from Bonferroni’s method—which is known as a more stringent method for multiple testing correction—were considered significant.

Heterogeneity in transcriptome-detecting platforms and patients’ treatment options may have existed. However, since overlapping results from two different platforms were used to determine significant findings, the platforms themselves appeared to have no specific impact on the results. Additionally, although patients received different treatment modalities, most samples were likely collected before the administration of chemotherapy or radiation. Even so, the clonality reflecting IDH status would not be expected to change significantly after treatment unless sampling occurred late during follow-up. As the primary goal of this study was to identify transcriptomic changes associated with IDH status, treatment options did not appear to have a significant effect on the analysis outcomes. Although there might be sample heterogeneity between the datasets, it seemed to have little effect on this analysis because many overlapping genes were found in the DEG and DCE analyses.

In the DEG analysis, GSE107850 yielded a small number of significant results when the Bonferroni correction was applied for multiple testing. This was likely due to the relatively small sample size and the strictness of the correction method. To identify more overlapping genes between datasets, the Benjamini-Hochberg (BH) method—which uses a less stringent threshold for multiple testing correction—was applied. The DEG list contained many genes likely to be involved in the pathophysiology of IDH mutation. The myogenic Differentiation 1 (*MYOD1*) gene plays a role in muscle development and differentiation and was the most upregulated gene in the IDH mutation group. Interestingly, *MYOD1* is known to be associated with the glioma subtype defined by different methylation profiles [34]. Another study reported the predictability of *MYOD1* for patient survival [35]. *LHX5*, one of the top-ranked upregulated genes, was associated with the prognosis of glioblastoma multiforme [36]. Transthyretin (*TTR*) was the most downregulated of the downregulated genes. Although there was no direct evidence of an association between *TTR* and LGG, the gene might be involved in the prognostic change of IDH mutation because TTR has a role in the transport of thyroid hormone related to the progression of glioma tumor cells [37]. The downregulation of *TTR* in the IDH mutation group may be linked to lower uptake of thyroid hormone, which causes a favorable prognosis through repression of tumor cell growth or differentiation.

The enrichment analysis of the common DEGs revealed many biological processes that might be clues for the underlying biology of the IDH mutation. In the enrichment analysis with GO BPs, terms related to organ formation, such as anatomical structure morphogenesis, anatomical structure development, developmental process, and system development, showed top-ranked significance (Table S5). Moreover, the term ‘signaling pathways regulating pluripotency of stem cells’ of the KEGG database was significant, which indicated that stem cell-like behaviors were active in the IDH mutation group. This is consistent with previous findings that IDH mutation in glioma stem cells (GSCs) attenuates poor prognosis [38] and is closely related to the maintenance of GSCs [1,39]. Considering that top-ranked TFs, including WT1, Sp1, and e2F-3, are related to stem cell proliferation and differentiation [40,41,42], transcriptomic changes in IDH mutation seem to be closely associated with the stemness of LGGs, which leads to differential prognosis [43].

In the DCE analysis, only seven gene pairs overlapped between the datasets (Table 1). Among them, the *CLDN11*–*TPPP3* gene pair may represent a potential prognostic marker associated with IDH mutation status. Upregulation of *TPPP3* has been linked to increased proliferation and invasion in glioblastoma cell lines [44], and *CLDN11* has been reported to enhance invasiveness in small cell lung cancer [45]. These findings suggest that this gene pair may be involved in molecular mechanisms associated with an unfavorable prognosis. Although the direction of co-expression changes differed between datasets, the pair still appears relevant to LGG pathophysiology, considering tumor heterogeneity and previous studies. For example, the Myelin Basic Protein (*MBP*)—ribonuclease A family member 1, pancreatic (*RNASE1*) gene pair is well-known for its dysregulation in LGGs. MBP is downregulated in oligodendrogliomas [46], with expression levels showing high variability across different LGG cell types [46]. *RNASE1* is also known as *RAC1* or *RNS1*. *RAC1* has been found to play a critical role in IDH-mutant LGGs [47]. Moreover, *MBP* has been shown to reduce phagocytosis in Sertoli cells by suppressing *RAC1* [48], and it is downregulated in IDH-mutant LGGs. These findings suggest that *MBP* downregulation may activate phagocytosis via *RAC1* in IDH-mutant LGGs. Although the number of overlapping gene pairs was small, the results were statistically significant even with the strict multiple testing correction of Bonferroni’s method. Moreover, when the genes from the significant results were considered, many TF gene sets were found to be significant (Table S7). Considering that the DCE analysis provides information about gene regulatory relationships, these results seem to be valuable information for understanding the pathophysiology of IDH mutations in LGGs.

In the results, significant terms in the enrichment analysis were related to glioma pathophysiology. For example, “synapse organization” is a GO BP term with genes related to synapse assembly, maturation, and pruning. Since synaptic communication and increased activities can increase tumor growth and glioma cell invasion, this term is consistent with the pathophysiology of gliomas. “Extracellular matrix organization” showed the most significant result in the enrichment analysis of CL3 genes (Table 2). Gliomas reshape the ECM, which increases angiogenesis, invasion, and therapeutic resistance, and leads to a poor prognosis [49]. The stem cell pluripotency-related terms were associated with self-renewal, consequent tumorigenesis [50], and resistance to therapy [51]. Considering the KLF4-regulated genes were significantly enriched in the DCE analysis results, stem cell pluripotency appears to be involved in the pathophysiology of IDH mutations and subsequent prognostic changes.

Several TFs that were significantly enriched in both datasets were previously reported to have functional roles. For example, GKLF, also known as KLF4, showed a highly significant result and is known to be involved in the self-renewal of glioma stem cells and glioma-genesis through its interaction with ITGB4 [52]. BTEB3 (also known as PTBP3) also showed strong enrichment and has been reported to promote glioblastoma tumorigenesis [53]. Although previous studies have not identified a specific role for ZNF148 in LGGs, its involvement in various other cancer types has been reported [54,55,56,57]. Notably, ZNF148 has been suggested as a potential regulator of TERT [54,55], which is associated with the prognosis of glioma patients [58]. These findings indicate that the commonly enriched TFs may provide insights into the molecular mechanisms underlying LGGs.

In addition to identifying common genes without considering the direction of co-expression changes between IDH statuses, gene pairs with the same direction of co-expression change were also analyzed. These pairs included cases in which one of the two *p*-values showed nominal significance (*p* < 0.05), and the other was significant after multiple testing correction. In total, 691 gene pairs were identified, comprising 474 unique genes (Table S14). When enrichment analysis was performed, the results were similar to those obtained from the common genes of significant DCE gene pairs, regardless of the direction of co-expression change (Table S15). For example, GO terms such as ‘system development,’ ‘synaptic signaling,’ and ‘neurogenesis’ were significantly enriched in both analyses. Other functional categories also showed similar enrichment patterns.

In the enrichment analysis of significant genes in the DCE analysis, more significant results (*n* = 1035) were found than in the analysis of DEGs (*n* = 377). This difference is interesting because the number of genes from the DCE analysis was less than that of the DEG analysis, and suggests that the DCE analysis can provide more information about gene regulatory relationships. While DEG analysis is one of the fundamental methods for genomics data analysis and can provide information about biomarkers, it provides no direct information about such relationships, which could lead to a difference in the number of significant results. Thus, finding similar biological processes in the enrichment analysis of DCE gene pairs is particularly relevant. For example, general processes such as ‘system development,’ ‘nervous system development,’ and ‘multicellular organism development,’ which are involved in organ development, were found to be significant (Table S6). Notably, the number of significant GO molecular function (MF) and GO cellular component (CC) terms was far greater than that of the enrichment analysis with DEGs, which indicated that genetic regulatory changes could occur without substantial changes in the magnitude of gene expression. It is also interesting that many seizure-related terms such as ‘focal impaired awareness seizure’ (*p* = 2.30 × 10^−6^), ‘epileptic encephalopathy’ (*p* = 3.40 × 10^−5^), and ‘focal-onset seizure’ (*p* = 1.67 × 10^−3^) were significant, which is consistent with the clinical finding that LGGs with IDH1 mutation tend to have seizures and can be related to patient survival [59]. The genes that are members of seizure-related gene sets were associated with seizures in gliomas (Table S6). For example, *GABRD* showed upregulation, while *GRIN2* was downregulated in LGG patients with seizures [60]. Additionally, *LGI1*, which has tumor suppressor activity in glioma, showed an epileptogenic effect [61]. *FGFR3* fusion was identified in diffuse lower-grade gliomas that have characteristics of long-term epilepsy-associated neuroepithelial tumors (LEAT) [62]. Considering that IDH mutations are known to induce epilepsy in gliomas, the genes in the DCE analysis results are worthy of further investigation for their relationship with epilepsy in gliomas.

In the MCL analysis of overlapping genes between the DEG and DCE results, 59 clusters were defined based on the human protein–protein interaction (PPI) network. While most clusters contained only a few genes (1–3 genes), the top-ranked clusters included a greater number of genes and showed significant enrichment results. Cluster 1 (CL1) contained 10 genes and exhibited many enrichment results (Table S10). The most significant Gene Ontology Biological Process (GO BP) term was ‘positive regulation of cell migration’ (FDR = 1.20 × 10^−3^), which appears to be associated with LGG prognosis. For example, SPI1 is known to promote glioma cell proliferation [63] and is associated with mesenchymal glioma stem cells, which exhibit the most malignant behavior [64]. *CD37,* another gene in the cluster, was upregulated in IDH wild-type gliomas and linked to poor prognosis [65]. These findings suggest that the genes in CL1 may play a role in determining patient prognosis. Cluster 2 (CL2) showed significant GO terms related to neural synapse function, such as ‘synapse organization’ (*p* = 1.60 × 10^−3^) and ‘neurotransmitter secretion (*p* = 1.45 × 10^−2^). In addition to GO terms, other functional categories also showed similar results (Table S11). Notably, *GABRB3*, a gene in CL2, has previously been identified as a survival biomarker in a study using the same TCGA LGG dataset, suggesting that the genes in CL2 may contribute to a distinct pathway involved in LGG prognosis due to their tight connectivity in the PPI network [66]. Cluster 3 (CL3) showed significant enrichment in ‘extracellular matrix organization’ (FDR = 1.33 × 10^−7^, Table 2), and previous studies have shown that the extracellular matrix plays a critical role in LGG progression [67,68].

The primary goal of this research was to identify transcriptomic changes associated with IDH status. If such changes are meaningful, classification performance based on them should be substantial. The results showed that classifiers using gene expression data achieved near-perfect performance, and this was replicated even when different models and datasets were applied. Although such models may be difficult to apply in practice for determining IDH status, the performance results provide further evidence that IDH status is strongly associated with significant transcriptomic changes. The classification performance revealed that IDH mutation status had a distinct molecular signature. Given that the datasets of the current research used different platforms, only the list of genes was used to build the classification model in the other dataset. However, even though different models were used in the classification, the prediction performance was excellent, and the AUCs of the models were almost perfect. In addition, LASSO with cross-validation also showed comparable results (Figures S3 and S4). This indicates that the genes selected by the LASSO model may be key components of the molecular signature involved in the pathophysiology of IDH mutation.

Because the IDH mutation group showed a better prognosis in LGGs, the perturbagen that induces a similar molecular signature to the IDH mutation group should be selected for therapeutic purposes. Based on this assumption, similar scores were chosen for perturbations (drugs) that showed positive results. The drug repurposing analysis using the CLUE platform identified NVP-TAE684, which was found to have a therapeutic effect on glioma. This compound is an anaplastic lymphoma kinase (ALK) inhibitor and has been associated with anti-tumor effects [69] and increased chemosensitivity, including in glioma cells [70]. Although its primary target is glioblastoma multiforme, other ALK inhibitors have also shown therapeutic potential [71]. In the previous research, a case of infant-type hemispheric glioma with a novel fusion gene, *SOX5::ALK*, was reported [72]. The patient received initial chemotherapy, surgery, and an ALK tyrosine kinase inhibitor (lorlatinib), which led to a reduction in tumor size and a partial response. While some ALK inhibitors have been shown to cause cell death in glioblastoma cell lines, there are no definite clinical studies of ALK inhibitors for gliomas [73]. Considering the promising results in small-cell lung cancers, the application of ALK inhibitors deserves to be tried. These results suggest that ALK inhibitors may represent a potential treatment option for LGG. The bisindolylmaleimide also reached the threshold value, but showed a marginal score. This chemical is known to have protein kinase C inhibitor activity and to suppress a glioma cell line [74]. In a previous clinical study, enzastaurin, a protein kinase C inhibitor, showed no definite improvement in the survival rate for glioblastomas, despite a Phase 2 study of recurrent glioblastomas demonstrating some therapeutic activity [75]. Although not a potential candidate compared to NVP-TAE684, bisindolylmaleimide might be worth investigating in future research on glioma.

## 5. Conclusions

In this research, DEG and DCE analyses identified significantly expressed genes and co-expressed gene pairs associated with the IDH mutation of LGG. Many biological processes and pathways involved in the differential prognosis of IDH mutation were identified through the enrichment analysis of the result genes from DEG and DCE analysis. Moreover, drugs that could be applied to treat LGGs were selected using these results. These results will guide future research in the search for potential therapeutic agents that can be applied in further clinical trials.

## Figures and Tables

**Table 1:** The overlapping gene pairs in the DCE analysis results of TCGA and GSE107850 data.

	TCGA			GSE107850				
Gene Pair	IDHwt	IDHmut	Pval	ID1	ID2	IDHwt	IDHmut	Pval
**STMN2_SVOP**	0.93	0.75	7.38 × 10^−10^	ILMN_1795679	ILMN_1764780	0.97	0.62	4.91 × 10^−6^
STAB1_COL6A3	0.79	0.26	3.65 × 10^−12^	ILMN_1655987	ILMN_2307861	−0.76	0.34	1.53 × 10^−5^
**CLDN11_TPPP3**	0.24	0.81	5.66 × 10^−14^	ILMN_1754103	ILMN_1797744	−0.56	0.67	3.36 × 10^−6^
**PPP2R2C_FGF12**	0.70	0.16	1.39 × 10^−9^	ILMN_1680507	ILMN_1672229	0.93	0.12	1.03 × 10^−6^
**CLDN11_DSCAML1**	0.56	−0.13	6.30 × 10^−11^	ILMN_1754103	ILMN_2075962	0.89	0.05	9.11 × 10^−6^
STAB1_CFH	0.78	0.29	3.98 × 10^−10^	ILMN_1655987	ILMN_1657803	−0.88	0.14	1.40 × 10^−6^
MBP_RNASE1	0.30	0.79	2.61 × 10^−11^	ILMN_2331544	ILMN_2333670	0.97	0.53	3.36 × 10^−6^

The values in the table represent IDH group-specific correlation coefficients indicating co-expression. Bold font indicates that the direction of co-expression change is consistent between datasets. TCGA: The Cancer Genome Atlas, DCE: differential co-expression, Pval: *p* value, ID: Illumina microarray probe identifier, IDHwt: wild-type isocitrate dehydrogenase, IDHmt: mutant IDH.

**Table 2:** Top-ranked terms in cluster 3 (CL3) from Markov clustering of common genes between the results of DEG and DCE analysis.

Term ID	Term Description	False Discovery Rate
GO:0030198	Extracellular matrix organization	1.33 × 10^−7^
GO:0005201	Extracellular matrix structural constituent	4.99 × 10^−10^
GO:0062023	Collagen-containing extracellular matrix	1.67 × 10^−7^
CL:19457	Extracellular matrix organization	2.99 × 10^−9^
hsa04974	Protein digestion and absorption	3.66 × 10^−6^
HSA-1474244	Extracellular matrix organization	3.05 × 10^−8^
WP4789	MED and pseudoachondroplasia genes	0.0017
HP:0003272	Abnormal hip bone morphology	0.00052
DOID:0070298	Multiple epiphyseal dysplasia 2	0.0035
BTO:0000421	Connective tissue	0.0038
GOCC:0005581	Collagen trimer	5.01 × 10^−7^
KW-0176	Collagen	1.93 × 10^−6^
PF01391	Collagen triple helix repeat (20 copies)	6.32 × 10^−6^
IPR008160	Collagen triple helix repeat	2.84 × 10^−5^

Table S12 shows the total results of the enrichment analysis. The analysis was performed in the STRING database.

## Data Availability

The original data presented in the study are openly available in CBioPortal (https://www.cbioportal.org, accessed on 23 April 2025) and the GEO database (https://www.ncbi.nlm.nih.gov/geo/, accessed on 17 April 2025).

## Supplementary Materials

The following supporting information can be downloaded at: https://www.mdpi.com/article/10.3390/biomedicines13092263/s1, Figure S1: result of LASSO analysis for predicting IDH mutation status in TCGA LGG data; Figure S2: result of LASSO analysis for predicting IDH mutation status in GSE107850; Figure S3: Result of LASSO regression with 10-fold cross-validation; Figure S4: Result of LASSO regression with 5-fold cross-validation; Figure S5: Overall analysis flow; Table S1: summary of clinical information; Table S2: DEG list of TCGA LGG data; Table S3: DEG list of GSE107850; Table S4: intersection of DEG analysis results from the two datasets; Table S5: enrichment analysis result of common DEG list; Table S6; enrichment analysis result of genes from significant DCE results; Table S7: common TFs from independent enrichment analysis of DCE results; Table S8: enrichment analysis result of overlapping genes from the results of DEG and DCE analysis; Table S9: cluster descriptions of MCL result; Table S10: enrichment analysis result of cluster 1 genes; Table S11: enrichment analysis result of cluster 2 genes; Table S12: enrichment analysis result of cluster 3 genes; Table S13: Selected predictor genes of LASSO analysis with TCGA LGG data; Table S14: Selected predictor genes of LASSO analysis with GSE107850 data; Table S15: enrichment analysis of DCE analysis result having the same direction of co-expression changes.

## References

[1] Zhang Y., Liu Y., Lang F., Yang C. (2022). IDH mutation and cancer stem cell. Essays Biochem..

[2] Youssef G., Miller J.J. (2020). Lower Grade Gliomas. Curr. Neurol. Neurosci. Rep..

[3] Fried I., Tabori U., Tihan T., Reginald A., Bouffet E. (2013). Optic pathway gliomas: A review. CNS Oncol..

[4] Evans L., Trinder S., Dodgshun A., Eisenstat D.D., Whittle J.R., Hansford J.R., Valvi S. (2024). IDH-mutant gliomas in children and adolescents—From biology to clinical trials. Front. Oncol..

[5] De Simone M., Choucha A., Ranalli C., Pecoraro G., Appay R., Chinot O.L., Dufour H., Iaconetta G. (2025). Astrocytomas IDH-mutant of posterior cranial fossa, clinical presentation, imaging features and onco-functional balance in surgical management. Neurosurg. Rev..

[6] Han S., Liu Y., Cai S.J., Qian M., Ding J., Larion M., Gilbert M.R., Yang C. (2020). IDH mutation in glioma: Molecular mechanisms and potential therapeutic targets. Br. J. Cancer.

[7] Xiang X., Liu Z., Zhang C., Li Z., Gao J., Zhang C., Cao Q., Cheng J., Liu H., Chen D. (2021). IDH Mutation Subgroup Status Associates with Intratumor Heterogeneity and the Tumor Microenvironment in Intrahepatic Cholangiocarcinoma. Adv. Sci. (Weinh).

[8] Raineri S., Mellor J. (2018). IDH1: Linking Metabolism and Epigenetics. Front. Genet..

[9] Park J.W., Turcan S. (2019). Epigenetic Reprogramming for Targeting IDH-Mutant Malignant Gliomas. Cancers.

[10] Unruh D., Zewde M., Buss A., Drumm M.R., Tran A.N., Scholtens D.M., Horbinski C. (2019). Methylation and transcription patterns are distinct in IDH mutant gliomas compared to other IDH mutant cancers. Sci. Rep..

[11] Mo H., Magaki S., Deisch J.K., Raghavan R. (2022). Isocitrate Dehydrogenase Mutations Are Associated with Different Expression and DNA Methylation Patterns of OLIG2 in Adult Gliomas. J. Neuropathol. Exp. Neurol..

[12] Brat D.J., Aldape K., Colman H., Holland E.C., Louis D.N., Jenkins R.B., Kleinschmidt-DeMasters B.K., Perry A., Reifenberger G., Stupp R. (2018). cIMPACT-NOW update 3: Recommended diagnostic criteria for “Diffuse astrocytic glioma, IDH-wildtype, with molecular features of glioblastoma, WHO grade IV”. Acta Neuropathol..

[13] Wu C., Song H., Fu X., Li S., Jiang T. (2020). Transcriptomic Analysis of Glioma Based on IDH Status Identifies ACAA2 as a Prognostic Factor in Lower Grade Glioma. Biomed. Res. Int..

[14] Li T., Yang Z., Li H., Zhu J., Wang Y., Tang Q., Shi Z. (2022). Phospholipase Cgamma1 (PLCG1) overexpression is associated with tumor growth and poor survival in IDH wild-type lower-grade gliomas in adult patients. Lab. Investig..

[15] Cai J., Hu Y., Ye Z., Ye L., Gao L., Wang Y., Sun Q., Tong S., Yang J., Chen Q. (2022). Immunogenic cell death-related risk signature predicts prognosis and characterizes the tumour microenvironment in lower-grade glioma. Front. Immunol..

[16] Ceccarelli M., Barthel F.P., Malta T.M., Sabedot T.S., Salama S.R., Murray B.A., Morozova O., Newton Y., Radenbaugh A., Pagnotta S.M. (2016). Molecular Profiling Reveals Biologically Discrete Subsets and Pathways of Progression in Diffuse Glioma. Cell.

[17] Gao Y., Weenink B., van den Bent M.J., Erdem-Eraslan L., Kros J.M., Sillevis Smitt P., Hoang-Xuan K., Brandes A.A., Vos M., Dhermain F. (2018). Expression-based intrinsic glioma subtypes are prognostic in low-grade gliomas of the EORTC22033-26033 clinical trial. Eur. J. Cancer.

[18] Kolberg L., Raudvere U., Kuzmin I., Vilo J., Peterson H. (2020). gprofiler2—An R package for gene list functional enrichment analysis and namespace conversion toolset g:Profiler. F1000Research.

[19] Harris M.A., Clark J., Ireland A., Lomax J., Ashburner M., Foulger R., Eilbeck K., Lewis S., Marshall B., Mungall C. (2004). The Gene Ontology (GO) database and informatics resource. Nucleic Acids Res..

[20] Kanehisa M., Goto S., Sato Y., Furumichi M., Tanabe M. (2012). KEGG for integration and interpretation of large-scale molecular data sets. Nucleic Acids Res..

[21] Milacic M., Beavers D., Conley P., Gong C., Gillespie M., Griss J., Haw R., Jassal B., Matthews L., May B. (2024). The Reactome Pathway Knowledgebase 2024. Nucleic Acids Res..

[22] Agrawal A., Balci H., Hanspers K., Coort S.L., Martens M., Slenter D.N., Ehrhart F., Digles D., Waagmeester A., Wassink I. (2024). WikiPathways 2024: Next generation pathway database. Nucleic Acids Res..

[23] Gargano M.A., Matentzoglu N., Coleman B., Addo-Lartey E.B., Anagnostopoulos A.V., Anderton J., Avillach P., Bagley A.M., Bakstein E., Balhoff J.P. (2024). The Human Phenotype Ontology in 2024: Phenotypes around the world. Nucleic Acids Res..

[24] Szklarczyk D., Kirsch R., Koutrouli M., Nastou K., Mehryary F., Hachilif R., Gable A.L., Fang T., Doncheva N.T., Pyysalo S. (2023). The STRING database in 2023: Protein-protein association networks and functional enrichment analyses for any sequenced genome of interest. Nucleic Acids Res..

[25] Musa A., Ghoraie L.S., Zhang S.D., Glazko G., Yli-Harja O., Dehmer M., Haibe-Kains B., Emmert-Streib F. (2018). A review of connectivity map and computational approaches in pharmacogenomics. Brief. Bioinform..

[26] Subramanian A., Narayan R., Corsello S.M., Peck D.D., Natoli T.E., Lu X., Gould J., Davis J.F., Tubelli A.A., Asiedu J.K. (2017). A Next Generation Connectivity Map: L1000 Platform and the First 1,000,000 Profiles. Cell.

[27] Gao J., Aksoy B.A., Dogrusoz U., Dresdner G., Gross B., Sumer S.O., Sun Y., Jacobsen A., Sinha R., Larsson E. (2013). Integrative analysis of complex cancer genomics and clinical profiles using the cBioPortal. Sci. Signal..

[28] Hutter C., Zenklusen J.C. (2018). The Cancer Genome Atlas: Creating Lasting Value beyond Its Data. Cell.

[29] Li B., Dewey C.N. (2011). RSEM: Accurate transcript quantification from RNA-Seq data with or without a reference genome. BMC Bioinform..

[30] IlluminaHumanv4.db: Illumina HumanHT12v4 Annotation Data (Chip illuminaHumanv4). https://bioconductor.org/packages/release/data/annotation/html/illuminaHumanv4.db.html.

[31] Cancer Genome Atlas Research N., Brat D.J., Verhaak R.G., Aldape K.D., Yung W.K., Salama S.R., Cooper L.A., Rheinbay E., Miller C.R., Vitucci M. (2015). Comprehensive, Integrative Genomic Analysis of Diffuse Lower-Grade Gliomas. N. Engl. J. Med..

[32] Tibshirani R. (1996). Regression Shrinkage and Selection Via the Lasso. J. R. Stat. Soc.Ser. B (Methodol.).

[33] Li Y., Ge X., Peng F., Li W., Li J.J. (2022). Exaggerated false positives by popular differential expression methods when analyzing human population samples. Genome Biol..

[34] Uhlmann K., Rohde K., Zeller C., Szymas J., Vogel S., Marczinek K., Thiel G., Nurnberg P., Laird P.W. (2003). Distinct methylation profiles of glioma subtypes. Int. J. Cancer.

[35] Ren P., Wang J.Y., Zeng Z.R., Li N.X., Chen H.L., Peng X.G., Bhawal U.K., Guo W.Z. (2022). A novel hypoxia-driven gene signature that can predict the prognosis and drug resistance of gliomas. Front. Genet..

[36] Ren P., Wang J., Li L., Lin X., Wu G., Chen J., Zeng Z., Zhang H. (2021). Identification of key genes involved in the recurrence of glioblastoma multiforme using weighted gene co-expression network analysis and differential expression analysis. Bioengineered.

[37] Davis F.B., Tang H.Y., Shih A., Keating T., Lansing L., Hercbergs A., Fenstermaker R.A., Mousa A., Mousa S.A., Davis P.J. (2006). Acting via a cell surface receptor, thyroid hormone is a growth factor for glioma cells. Cancer Res..

[38] Yao Q., Cai G., Yu Q., Shen J., Gu Z., Chen J., Shi W., Shi J. (2018). IDH1 mutation diminishes aggressive phenotype in glioma stem cells. Int. J. Oncol..

[39] Haddock S., Alban T.J., Turcan S., Husic H., Rosiek E., Ma X., Wang Y., Bale T., Desrichard A., Makarov V. (2022). Phenotypic and molecular states of IDH1 mutation-induced CD24-positive glioma stem-like cells. Neoplasia.

[40] Olszewski M., Huang W., Chou P.M., Duerst R., Kletzel M. (2005). Wilms’ tumor 1 (WT1) gene in hematopoiesis: A surrogate marker of cell proliferation as a possible mechanism of action?. Cytotherapy.

[41] Felthaus O., Viale-Bouroncle S., Driemel O., Reichert T.E., Schmalz G., Morsczeck C. (2012). Transcription factors TP53 and SP1 and the osteogenic differentiation of dental stem cells. Differentiation.

[42] Guetta-Terrier C., Karambizi D., Akosman B., Zepecki J.P., Chen J.S., Kamle S., Fajardo J.E., Fiser A., Singh R., Toms S.A. (2023). Chi3l1 Is a Modulator of Glioma Stem Cell States and a Therapeutic Target in Glioblastoma. Cancer Res..

[43] Loh J.J., Ma S. (2024). Hallmarks of cancer stemness. Cell Stem Cell.

[44] Xu X., Hou Y., Long N., Jiang L., Yan Z., Xu Y., Lv Y., Xiang X., Yang H., Liu J. (2023). TPPP3 promote epithelial-mesenchymal transition via Snail1 in glioblastoma. Sci. Rep..

[45] Sakamoto S., Inoue H., Takino T., Kohda Y., Yoshida J., Ohba S., Usami I., Suzuki T., Kawada M., Hatakeyama M. (2025). Claudin-11 Enhances Invasive and Metastatic Abilities of Small-Cell Lung Cancer Through MT1-MMP Activation. Cancer Sci..

[46] Dmytrenko V.V., Boiko O.I., Shostak K.O., Bilets’kyi A.V., Malysheva T.A., Shamaiev M.I., Kliuchka V.M., Rozumenko V.D., Zozulia Iu P., Kavsan V.M. (2009). Expression of myelin basic protein and glial fibrillary acidic protein genes in human glial brain tumors. Tsitol. Genet..

[47] Liu Y., Lu Y., Li A., Celiku O., Han S., Qian M., Yang C. (2020). mTORC2/Rac1 Pathway Predisposes Cancer Aggressiveness in IDH1-Mutated Glioma. Cancers.

[48] Gong P., Chen S., Zhang L., Hu Y., Gu A., Zhang J., Wang Y. (2018). RhoG-ELMO1-RAC1 is involved in phagocytosis suppressed by mono-butyl phthalate in TM4 cells. Environ. Sci. Pollut. Res. Int..

[49] Mohiuddin E., Wakimoto H. (2021). Extracellular matrix in glioblastoma: Opportunities for emerging therapeutic approaches. Am. J. Cancer Res..

[50] Hattermann K., Fluh C., Engel D., Mehdorn H.M., Synowitz M., Mentlein R., Held-Feindt J. (2016). Stem cell markers in glioma progression and recurrence. Int. J. Oncol..

[51] Mahdi A., Aittaleb M., Tissir F. (2025). Targeting Glioma Stem Cells: Therapeutic Opportunities and Challenges. Cells.

[52] Ma B., Zhang L., Zou Y., He R., Wu Q., Han C., Zhang B. (2019). Reciprocal regulation of integrin beta4 and KLF4 promotes gliomagenesis through maintaining cancer stem cell traits. J. Exp. Clin. Cancer Res..

[53] Xie P., Zhang Y., Chen R., Zheng J., Cui G. (2022). PTBP3 promotes tumorigenesis of glioblastoma by stabilizing Twist1. Transl. Oncol..

[54] Fang J., Jia J., Makowski M., Xu M., Wang Z., Zhang T., Hoskins J.W., Choi J., Han Y., Zhang M. (2017). Functional characterization of a multi-cancer risk locus on chr5p15.33 reveals regulation of TERT by ZNF148. Nat. Commun..

[55] Chua B.H., Zaal Anuar N., Ferry L., Domrane C., Wittek A., Mukundan V.T., Jha S., Butter F., Tenen D.G., Defossez P.A. (2023). E4F1 and ZNF148 are transcriptional activators of the -57A > C and wild-type TERT promoter. Genome. Res..

[56] Cheng S., Liu L., Wang D., Li Y., Li S., Yuan J., Huang S., Xu Z., Jia B., Li Z. (2023). Upregulation of the ZNF148/PTX3 axis promotes malignant transformation of dendritic cells in glioma stem-like cells microenvironment. CNS Neurosci. Ther..

[57] Gao X., Ma C., Sun X., Zhao Q., Fang Y., Jiang Y., Shen K., Shen X. (2020). Upregulation of ZNF148 in SDHB-deficient gastrointestinal stromal tumor potentiates Forkhead box M1-mediated transcription and promotes tumor cell invasion. Cancer Sci..

[58] Olympios N., Gilard V., Marguet F., Clatot F., Di Fiore F., Fontanilles M. (2021). TERT Promoter Alterations in Glioblastoma: A Systematic Review. Cancers.

[59] Fan X., Li Y., Shan X., You G., Wu Z., Li Z., Qiao H., Jiang T. (2018). Seizures at presentation are correlated with better survival outcomes in adult diffuse glioma: A systematic review and meta-analysis. Seizure.

[60] Lin H., Yang Y., Hou C., Huang Y., Zhou L., Zheng J., Lv G., Mao R., Chen S., Xu P. (2021). Validation of the functions and prognostic values of synapse-associated proteins in lower-grade glioma. Biosci. Rep..

[61] Gu W., Brodtkorb E., Piepoli T., Finocchiaro G., Steinlein O.K. (2005). LGI1: A gene involved in epileptogenesis and glioma progression?. Neurogenetics.

[62] Xie M., Wang X., Duan Z., Luan G. (2022). Low-grade epilepsy-associated neuroepithelial tumors: Tumor spectrum and diagnosis based on genetic alterations. Front. Neurosci..

[63] Du B., Gao W., Qin Y., Zhong J., Zhang Z. (2022). Study on the role of transcription factor SPI1 in the development of glioma. Chin. Neurosurg. J..

[64] Song Y., Zhang Y., Wang X., Han X., Shi M., Xu L., Yu J., Zhang L., Han S. (2024). SPI1 activates TGF-beta1/PI3K/Akt signaling through transcriptional upregulation of FKBP12 to support the mesenchymal phenotype of glioma stem cells. Brain Pathol..

[65] Yan X., Zhou Q., Zhu H., Liu W., Xu H., Yin W., Zhao M., Jiang X., Ren C. (2021). The clinical features, prognostic significance, and immune heterogeneity of CD37 in diffuse gliomas. iScience.

[66] Badalotti R., Dalmolin M., Malafaia O., Ribas Filho J.M., Roesler R., Fernandes M.A.C., Isolan G.R. (2024). Gene Expression of GABA(A) Receptor Subunits and Association with Patient Survival in Glioma. Brain Sci..

[67] Barbosa L.C., Machado G.C., Heringer M., Ferrer V.P. (2024). Identification of established and novel extracellular matrix components in glioblastoma as targets for angiogenesis and prognosis. Neurogenetics.

[68] Cheng X., Liu Z., Liang W., Zhu Q., Wang C., Wang H., Zhang J., Li P., Gao Y. (2023). ECM2, a prognostic biomarker for lower grade glioma, serves as a potential novel target for immunotherapy. Int. J. Biochem. Cell Biol..

[69] Li Y., Ye X., Liu J., Zha J., Pei L. (2011). Evaluation of EML4-ALK fusion proteins in non-small cell lung cancer using small molecule inhibitors. Neoplasia.

[70] Lorente M., Torres S., Salazar M., Carracedo A., Hernandez-Tiedra S., Rodriguez-Fornes F., Garcia-Taboada E., Melendez B., Mollejo M., Campos-Martin Y. (2011). Stimulation of the midkine/ALK axis renders glioma cells resistant to cannabinoid antitumoral action. Cell Death Differ..

[71] Kawauchi D., Takahashi M., Satomi K., Yamamuro S., Kobayashi T., Uchida E., Honda-Kitahara M., Narita Y., Iwadate Y., Ichimura K. (2021). The ALK inhibitors, alectinib and ceritinib, induce ALK-independent and STAT3-dependent glioblastoma cell death. Cancer Sci..

[72] Tsai C.C., Huang M.H., Fang C.L., Hsieh K.L., Hsieh T.H., Ho W.L., Chang H., Tsai M.L., Kao Y.C., Miser J.S. (2024). An Infant-Type Hemispheric Glioma With SOX5::ALK: A Novel Fusion. J. Natl. Compr. Canc. Netw..

[73] Kim J.H. (2021). Prognostic and predictive markers in glioblastoma and ALK overexpression. J. Pathol. Transl. Med..

[74] Morreale A., Mallon B., Beale G., Watson J., Rumsby M. (1997). Ro31-8220 inhibits protein kinase C to block the phorbol ester-stimulated release of choline- and ethanolamine-metabolites from C6 glioma cells: p70 S6 kinase and MAPKAP kinase-1beta do not function downstream of PKC in activating PLD. FEBS Lett..

[75] Wick W., Puduvalli V.K., Chamberlain M.C., van den Bent M.J., Carpentier A.F., Cher L.M., Mason W., Weller M., Hong S., Musib L. (2010). Phase III study of enzastaurin compared with lomustine in the treatment of recurrent intracranial glioblastoma. J. Clin. Oncol..

