# Supplementary material for: Transcriptomic Profile of Isocitrate Dehydrogenase Mutant Type of Lower-Grade Glioma Reveals Molecular Changes for Prognosis
Source: Biomedicines. 2025 Sep 14;13(9):2263. doi: 10.3390/biomedicines13092263 (PMC12467759; doi:10.3390/biomedicines13092263)
Supplement: Supplementary file 1 [file biomedicines-13-02263-s001.zip › Supplementary Results.pdf]

Supplementary Results

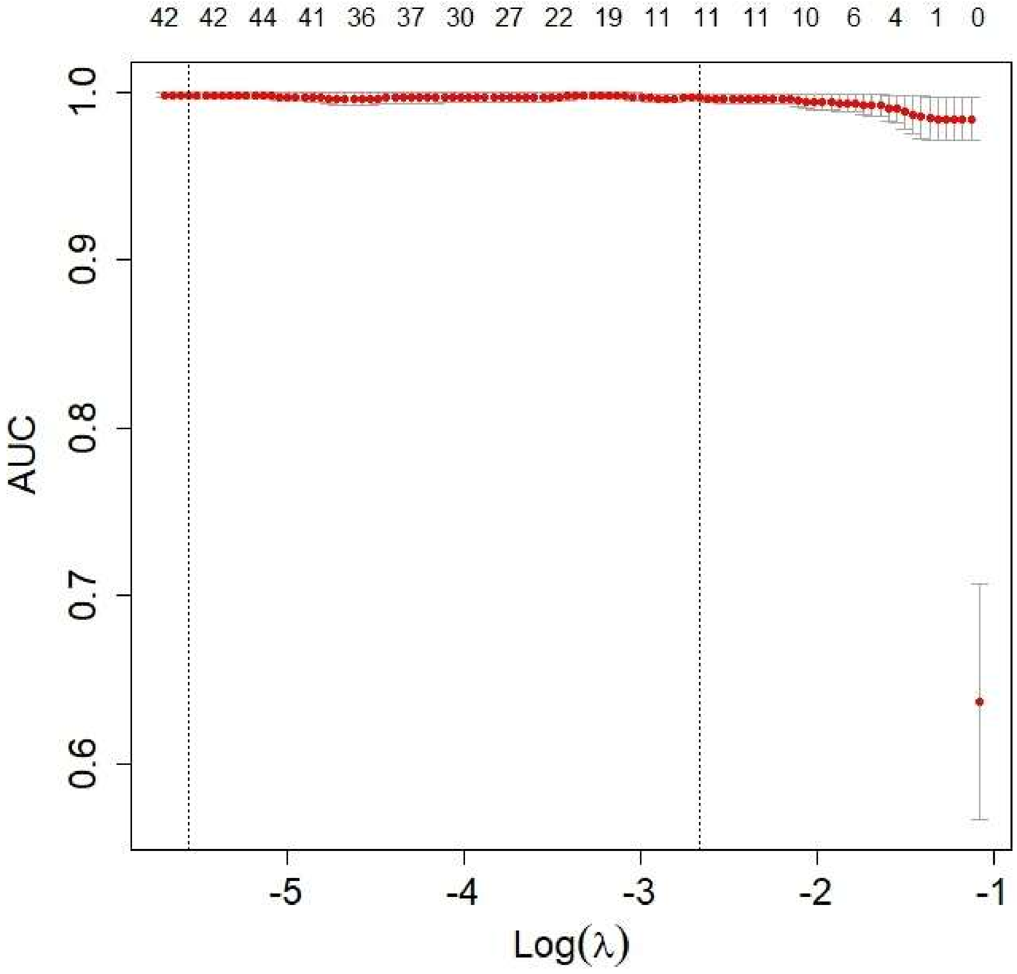

Figure S1. Result of LASSO cross-validation analysis for predicting IDH mutation status with TCGA data

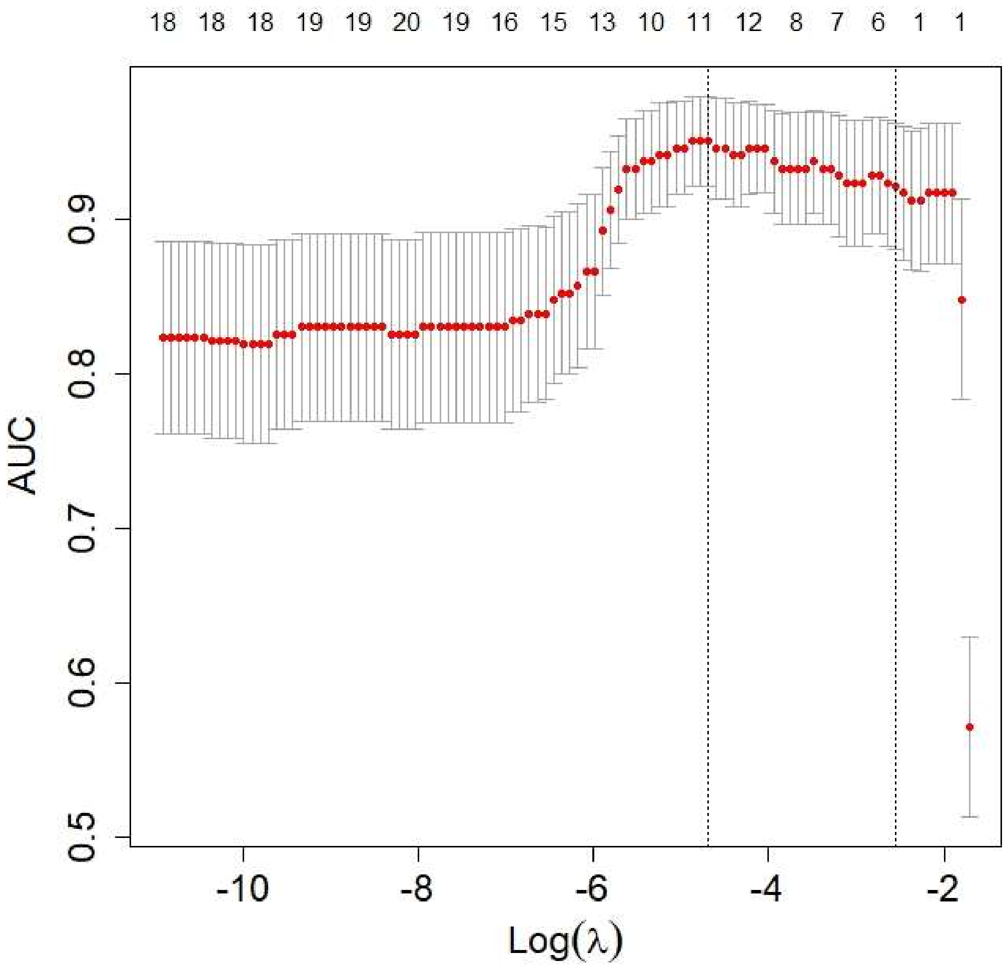

Figure S2. Result of LASSO cross-validation analysis for predicting IDH mutation status with GSE107850 data

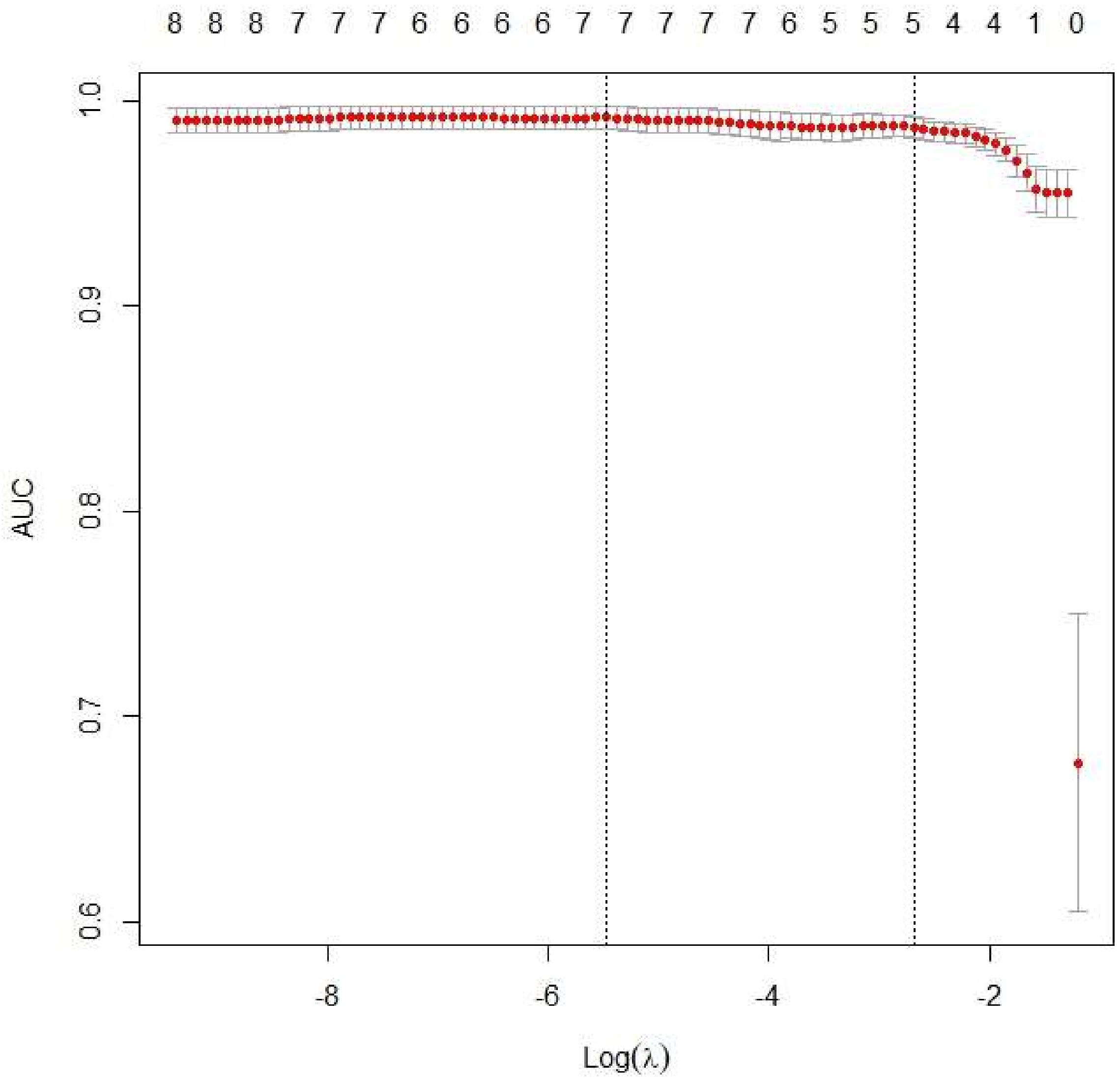

Figure S3. Result of LASSO regression with 10-fold cross-validation. The maximal mean prediction accuracy was obtained with a model with 7 genes.

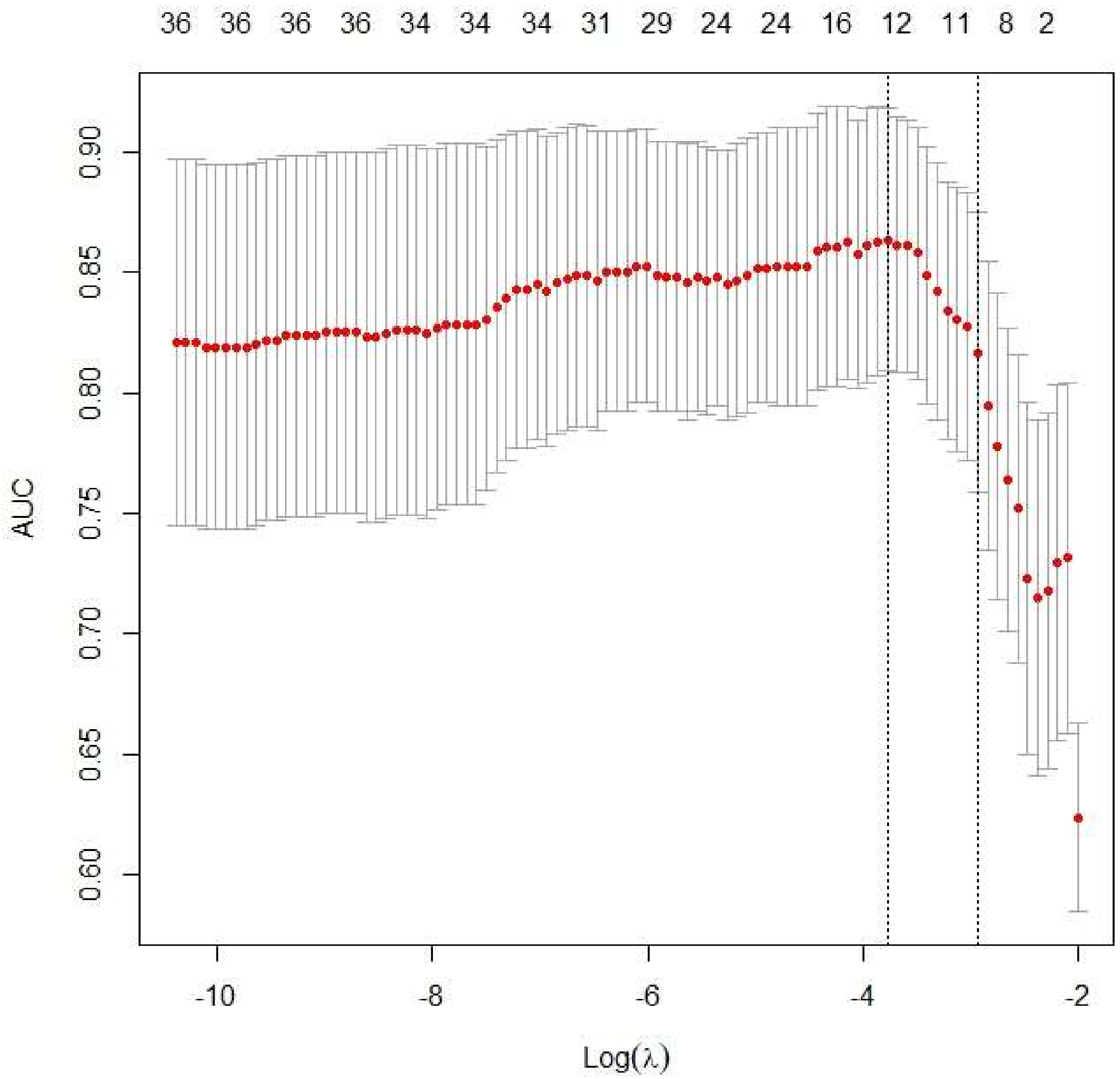

Figure S4. Result of LASSO regression with 5-fold cross-validation. Considering the unbalance class labels of GSE107850 data, 5-fold cross-validation was applied.

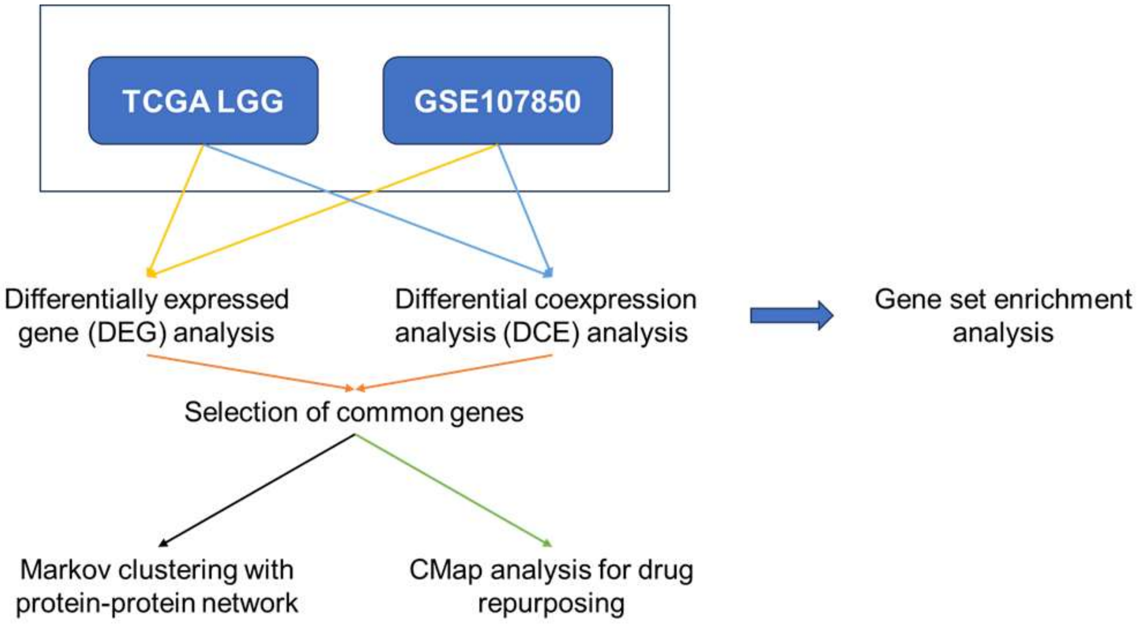

Figure S5. Overall analysis flow

Table S1. Summary of clinical information

Data	variables	IDH_wild	IDH_mutant	P value
**TCGA (IDH wt/mt = 92/415)**	Age	52.59 ± 14.13	40.85 ± 12.23	2.07E-11
	Sex (M/F)	52/40	230/184	0.88
	OS (+/-)	42/50	342/73	2.73E-13
	OS_time (months)	19.54 ± 26.44	34.14 ±	8.53E-06
**GSE107850 (IDH wt/mt = 14/166)**	Age	48.26 ± 13.15	43.43 ± 10.84	0.2292
	Sex (M/F)	11/3	89/77	0.1273
	OS (+/-)	10/4	79/87	0.1513
	OS_time (days)	748.57 ± 26.44	1061.79 ± 31.87	0.1195

Statistical test between IDH groups was performed with T test and chi-square test for continuous and categorical variables, respectively. OS; overall survival, IDH: isocitrate dehydrogenase, wt: wild type, mut: mutant

Table S13. Selected predictor genes of LASSO analysis with TCGA LGG data

Gene	beta
**ANXA5**	-7.09E-05
**AQP5**	-0.00158
**ARSD**	-6.25E-05
**ARSE**	-0.00077
**C9orf64**	-0.00066
**CACNG5**	-0.00424
**CCDC46**	-0.00013
**CD244**	-0.00633
**CD58**	-0.00075
**CDH4**	-0.00012
**CHRNB4**	-0.02319
**COL24A1**	-0.00196
**COLEC10**	-0.06052
**COX15**	0.000254
**CPA1**	-0.00966
**CUL7**	-8.33E-05
**EMP3**	-0.00027
**EVC**	-0.0004
**FAR2**	-0.00057
**FBLN7**	-0.00037
**FBXO17**	-0.00011
**GPR3**	-0.00243
**GSG1**	-0.07462
**HOXC10**	-6.24E-05
**KIAA0495**	-0.00636
**LPAR3**	-0.00697
**MAB21L1**	-0.00648
**MT1L**	-0.00223
**MYOZ3**	-0.00241
**NCRNA00092**	-0.00312
**NSUN7**	-0.00071
**NTNG1**	-0.00201
**PCDH11Y**	-0.00206
**SLC43A3**	-0.00101
**SP6**	-0.00242
**SPRY4**	-1.47E-05
**SWAP70**	-0.00081

**TAAR9**	-0.06766
**TFRC**	-0.00019
**TMEM159**	-0.00142
**VAV3**	-0.00046
**WEE1**	-0.0003

Table S14. Selected predictor genes of LASSO analysis with GSE107850 data

Gene	Gene Symbol	Beta
**ILMN_1669983**	RGS22	-1.60652
**ILMN_1670881**	CHST6	-0.0514
**ILMN_1696546**	FZD1	-1.20077
**ILMN_1707592**	RGN	-0.06013
**ILMN_1764364**	TAFA3	-0.2933
**ILMN_1772064**	NA	-1.00281
**ILMN_2090641**	FAM110C	-0.11296
**ILMN_2147517**	CD58	-0.02426
**ILMN_2179083**	LOXL4	-0.10454
**ILMN_3197097**	TSTD1	-0.56357
**ILMN_3245773**	GSAP	-0.82981
